# Crystal structure evolution of an energetic compound dihydroxylammonium 5,5′-bistetrazole-1,1′-diolate induced by solvents†

**DOI:** 10.1039/d0ra01182g

**Published:** 2020-03-26

**Authors:** Xin Xu, Dong Chen, Hongzhen Li, Mi Yan, Ying Xiong, Haixia Zhao, Rong Xu

**Affiliations:** Institute of Chemical Materials, China Academy of Engineering Physics Mianyang 621900 China xurwjy@caep.cn; College of Environment and Safety Engineering, North University of China Taiyuan 030051 China zhhx@nuc.edu.cn

## Abstract

Recently, energetic ionic salts have become a research hotspot due to their attractive properties, such as high density, high heat of formation, and environmental friendliness. Dihydroxylammonium 5,5′-bistetrazole-1,1′-diolate (TKX-50) is a typical nitrogen-rich energetic ionic salt, which has broad application prospects. However, the research on the stability and crystal structure evolution of TKX-50 in different solvent systems is insufficient. Herein, we investigated the crystal structure transformations and searched for new solid forms of TKX-50 under different conditions *via* a solvent induction method. The phase composition of all screened samples was analyzed by powder or single-crystal X-ray diffraction. Three new solid forms of [NH_2_(CH_3_)_2_^+^][BTO^−^], [NH_2_(CH_3_CH_2_)_2_^+^]_2_[BTO^2−^], [NHOH(CH_3_CCH_3_)^+^][BTO^−^] H_2_O were obtained from DMAC, DEF and AC/MT, respectively. Furthermore, the energetic properties were evaluated through EXPLO5.

## Abstract 2

Three new solvent compounds obtained by dihydroxylammonium 5,5′-bistetrazole-1,1′-diolate recrystallized from three solvents.

## Graphical abstract

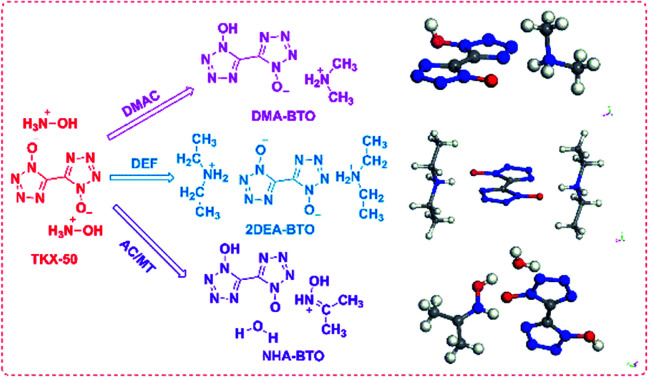

## Introduction

The solid form stability is one of the most important properties of explosives; different solid forms (such as solvates,^1^ polymorphs^2^ and salts^3^) will directly affect the safety, energy and storage life of weapons. In general, factors like solvents, temperature and pressure in crystallization can affect the crystalline solid forms of explosives due to the arisen changes in the structures of energetic molecules, and the transformed solid forms are irreversible in most cases. Among them, solvents act as the most important and influential role on the structure evolution in crystallization, but it has great unpredictability. During the crystallization process, some energetic compounds may react with the solvents to form solvates, salts or be induced to be a certain crystal polymorph. Unfortunately, these new solid forms hold serious drawbacks such as unstable structure, lower density and poor detonation performance, which have greatly limited their application. Up to now, the most used explosives' solid forms in the military are only as follows: α-RDX (hexahydro 1,3,5-trinitro-1,3,5-triazine),^4–7^ β-HMX (1,3,5,7-tetranitro-1,3,5,7-azacyclo-octane),^8–10^ ε-CL-20 (2,4,6,8-hexanitro-2,4,6,8,10,12-hexaazatetracyclododecane)^11,12^ and α-FOX-7 (1,1-diamino-2,2-dinitroethylene).^13–15^

In recent years, energetic ionic salts (EISs) which consist of energetic cations and anions have been regarded as promising alternatives to molecular explosives such as RDX and HMX due to their good properties including high density, excellent safety and benign heat of formation.^16–24^ However, it is worth noting that during the structural evolution of EISs induced by solvents, phase dissociation is an easily overlooked but important phenomenon compared to the possible polymorphs or solvent compounds. In phase dissociation, the primary multicomponent compound tend to dissociate to its unitary part, which can bring huge risks to explosives. In previous work, Li's^25^ group has verified the phase dissociation phenomenon of energetic ionic salt carbonic dihydrazidinium bis [3-(5-nitroimino-1,2,4-triazolate)] (CBNT) for the first time. However, there is few study on the solid form transformations of high energy explosives especially for EISs for now, in which the cause and rule of series phenomenon are still unclear.^26^ In order to further understand the physicochemical reliability of EISs, it is necessary to investigate their structure evolution and find the final solid forms in solvents induced crystallization process.

The energetic ionic salt dihydroxylammonium 5,5′-bistetrazole-1,1′-diolate (TKX-50) was first reported by Klapötke and coworkers^27^ in 2012. Then, extensive attention has pointed to its excellent properties such as high density of 1.918 g cm^−3^, high detonation velocity and pressure of 9698 ms^−1^ and 42.4 GPa, which showed great advantages and application prospects in the field of propellants. However, to the best of our knowledge, the previous research on TKX-50 was mainly focused on the analysis^28,29^ of synthetic methods and performance.^30–34^ In 2009, Li *et al.*^35^ found that TKX-50 remains stable in DMSO, deionized water, ethyl acetate, acetonitrile, methanol, ethanol, petroleum ether and hexane. It is remarkable that TKX-50 can react with DMF in different ways under different conditions, such as a wide temperature range from 25 °C to 150 °C. The products in reactions between TKX-50 and DMF are dimethylammonium 5,5′-bistetrazole-1-hydroxy-1′-oxygen (DMA-BTO), dimethylamine 5,5′-bistetrazole-1,1′-diolate (2DMA-BTO) and diammonium 5,5′-bistetrazole-1,1′-diolate (**ABTOX**). However, this work only studied the structural transformation of TKX-50 in limited pure solvents, and the result in binary solvent systems is unknown.

In this work, four different crystallization methods were used to study the crystal structure evolution of TKX-50 in eight different solvent systems. Four powder samples and three single crystal structures were obtained through experiments. On the basis of previous work, we performed further crystallization experiments on DMF homologs including DMAC and DEF solvents, and found that the single crystal obtained from DMAC has the same crystal structure as obtained from DMF. An interesting experimental phenomenon is that although TKX-50 can stably exist in pure solvent of acetone or methanol, a reaction occurred in their mixed solvent system. Moreover, the thermal and energy properties (standard molar enthalpy of formation, detonation velocity, and detonation pressure) of these new compounds were studied in detail.

## Experimental

### Solid form screening

The solubility of TKX-50 in common solvents was tested, including: dimethyl sulfoxide (DMSO), distilled water (H_2_O), *N*,*N*-diethyl methylformamide (DEF), *N*,*N*-dimethylacetamide (DMAC), *N*-methyl-2-pyrrolidone (NMP), 1,4-butyrolactone (BL), ethyl acetate ester (EA) and acetone (AC)/methyl alcohol (MT). The experimental process was as follows: added a certain amount of TKX-50 to distilled water in a three-necked flask, stirred at room temperature, finally filtered and calculated the solubility of TKX-50 in distilled water. Similarly, the solubility of TKX-50 in the remaining solvents was listed in the Table 1. Solvents with the solubility above 0.01 g/100 mL were continued to be screened in solid form.

**Table 1:** The solubility of TKX-50 in common solvents at 25 °C

Solubility	>0.5 g/100 mL	0.06 g/100 mL–0.5 g/100 mL	0.01 g/100 mL–0.06 g/100 mL	<0.01 g/100 mL
Solvent	DMSO	Water	DEF, DMAC, NMP, BL, AC/MT	EA

Seven solvents with the solubility higher than 0.01 g/100 mL were selected to screen possible TKX-50 solids by screening solutions in the literature.^36^ Added a certain amount of TKX-50 to a three-necked flask with distilled water, stirred and heated until TKX-50 completely dissolved and saturated. The resulting solution was evenly divided into four portions for processing in different ways. The first solution was quickly cooled to 0 °C by placing it in ice water and kept for 1 hour. The second solution was slowly cooled to room temperature in a ventilated place. The third part was covered with perforated plastic wrap and allowed to evaporate slowly. The fourth part was quickly evaporated using an air pump. Finally, the obtained precipitates were filtered, washed and dried.

### Single crystal X-ray diffraction (SC-XRD)

Suitable crystals were chosen and placed in a Rigaku supernova Single X-ray Diffractometer area detector using graphite monochromated Mo Kα radiation (*λ* = 0.71073 Å) at 298(2) K. Their structures were solved by direct methods and successive Fourier difference syntheses using the SHELXTL software suite30. Hydrogen atoms attached to oxygen were placed from difference Fourier maps and were refined using riding model. Data collection parameters and refinement statistics were listed in Table S1.†

### Powder X-ray diffraction (PXRD)

Powder X-ray diffraction patterns were recorded with the Cu Kα radiation (*λ* = 1.54056 Å). The current and voltage were set at 30 mA and 40 kV, respectively. The data were collected over the range from 5°to 50° with a step size of 0.02°.

### Differential scanning calorimetry (DSC) and thermogravimetric (TG) analysis

DSC and TG analysis measurements were performed with a METTLER TOLEDO TGA/DSC2 calorimeter at a scan rate of 10 °C min^−1^ under nitrogen atmosphere of 20 mL min^−1^.

## Results and discussion

### Solid form screening

In this paper, four different crystallization methods including quick cooling, slow cooling, quick evaporation, and slow evaporation were used to conduct screening experiments on the precipitation of TKX-50. The results were shown in Table 2.

**Table 2:** The screening results for TKX-50 in solution^a^

Solvent	Quick cooling	Slow cooling	Quick evaporation	Slow evaporation
DMSO	—	DMSO_P_	—	DMSO_P_
H_2_O	H_2_O_P_	H_2_O_P_	H_2_O_P_	H_2_O_P_
DMAC	—	—	DMAC_C_ (1)	—
DEF	—	—	DEF_C_ (2)	—
NMP	NMP_P_	NMP_P_	NMP_P_	NMP_P_
BL	BL_P_	BL_P_	BL_P_	BL_P_
AC/MT	—	—	—	AC/MT_C_ (3)

a —: no crystalline compound obtained, ∗P: powder, ∗C: single crystal.

**Fig. 1:**
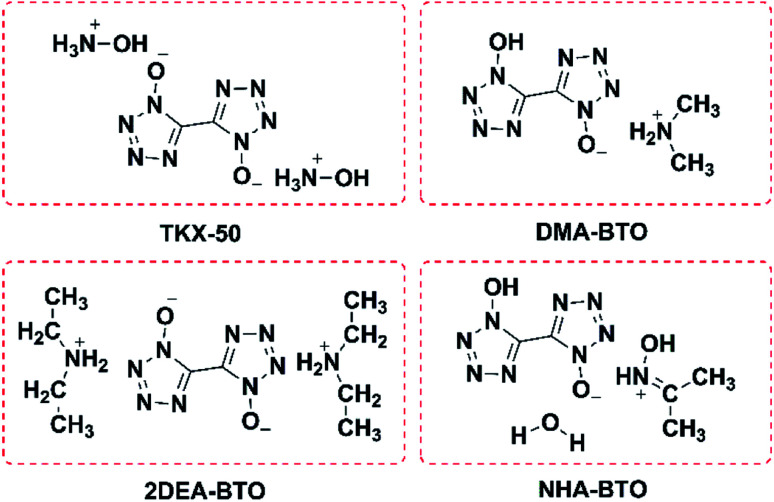
The molecular structure of TKX-50, DMA-BTO, 2DEA-BTO and NHA-BTO.

**Fig. 2:**
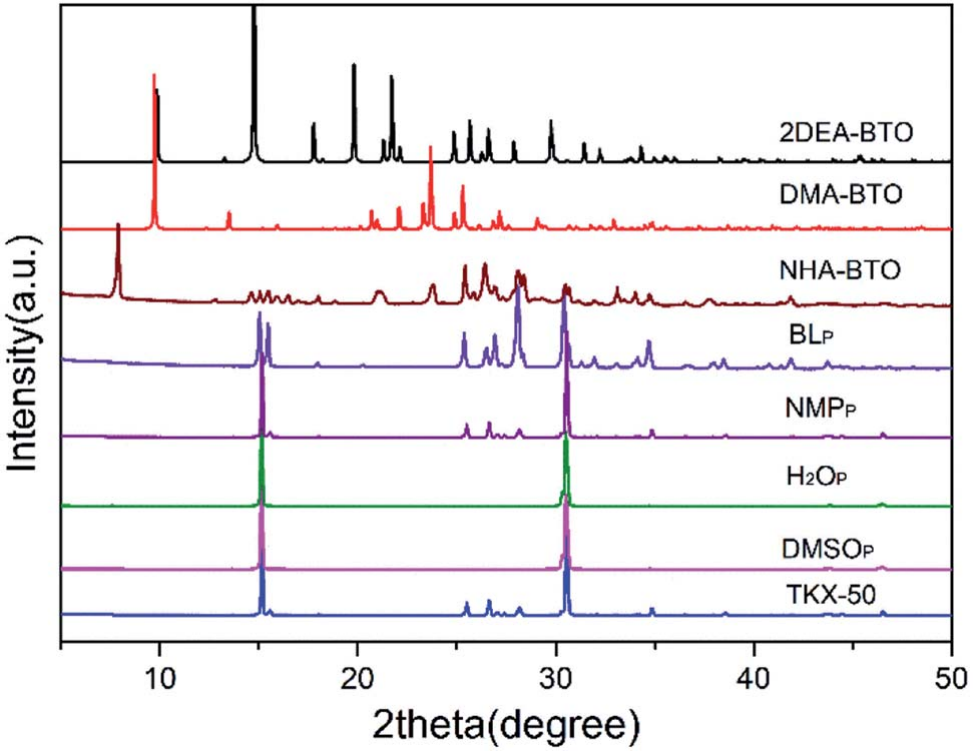
Powder diffraction patterns of TKX-50 and the results by experiment.

According to Table 2, different crystallization methods and solvent systems had a large effect on the solid form of TKX-50. In our experiment, five solid samples were obtained, and we analyzed three structures among them including DMAC_C_ ([NH_2_(CH_3_)_2_^+^][BTO^−^]), DEF_C_ ([NH_2_(CH_3_CH_2_)_2_^+^]_2_[BTO^2−^]), and AC/MT_C_ ([NHOH(CH_3_CCH_3_)^+^][BTO^−^]·H_2_O). The structure is shown in Fig. 1. As shown in Fig. 2, the PXRD patterns indicated that the solid samples obtained from different solvents had different crystal structures, which due to the forming of new compounds that TKX-50 crystallized in different solvents. For the DMAC solvent, the diffraction pattern of the obtained compound was completely different from the TKX-50 raw material, and we define the product as DMA-BTO. However, single crystal diffraction indicated that the product obtained from the DMAC solvent had the same crystal structure as the product obtained from the DMF solvent. In addition, the product obtained from the DEF solvent which PXRD spectrum observed some new sharp peaks in the 2*θ* range of 5–10°and 15–25°was defined as 2DEA-BTO. The product obtained in the mixed solvent of acetone/methanol was defined as NHA-BTO, which was also with the different diffraction pattern from that of the raw material. In short, the diffraction peaks of the solids obtained in DMSO, H_2_O, NMP, and BL were coincided with that of the raw material TKX-50, which indicated that the above five solvents could be used as solvents for recrystallization and process of TKX-50. In the studied solvent systems DEF, DMAC and mixed solvent of acetone/methyl alcohol, the crystal structure of TKX-50 may be dissociated and formed other new compounds, which may destroy its original stability, safety and performance. Therefore, during the recrystallization, synthesis and storage of TKX-50, special care should be taken to avoid selecting these solvents.

**Fig. 3:**
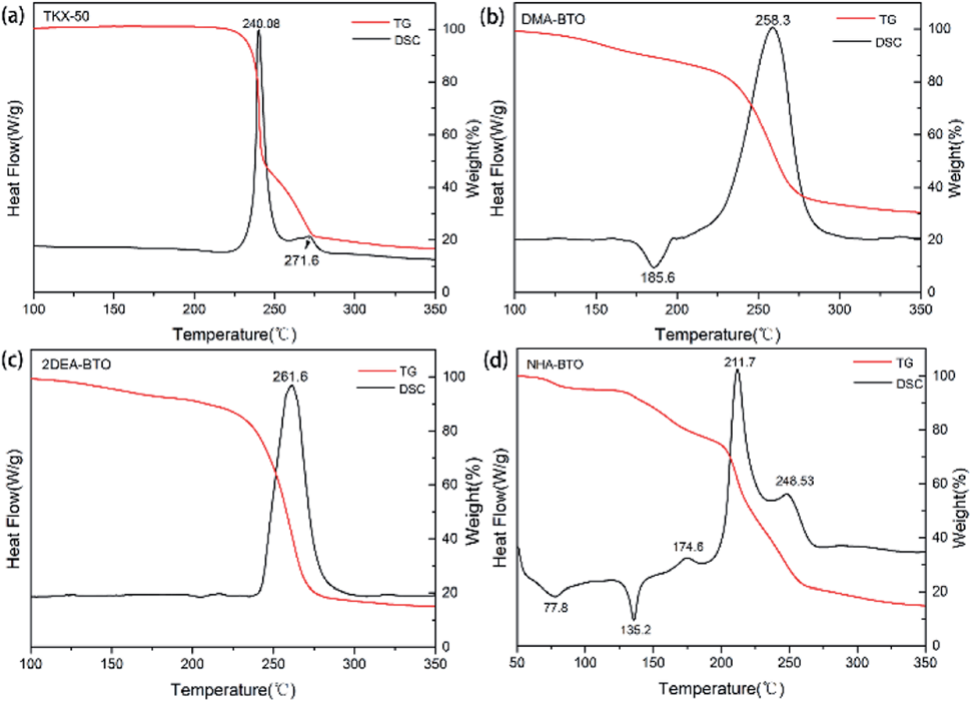
TG-DSC curves, (a) TKX-50; (b) DMA-BTO; (c) 2DEA-BTO; (d) NHA-BTO.

An important index for evaluating high-energy materials was thermal stability, the thermal properties directly affect their safety performance and applications. Therefore, the thermal properties of the raw materials TKX-50, DMA-BTO, 2DEA-BTO and NHA-BTO compounds were investigated *via* TG-DSC and the result curves were showed in Fig. 3. In general, the thermal behavior of the new compounds were significantly different from that of the raw materials. It could be seen from the curves that TKX-50 showed distributed decomposition with two exothermic peaks, the main exothermic decomposition temperature peak was at 240.3 °C. For compound DMA-BTO, the main decomposition temperature was at 258.3 °C, there was also an endothermic peak at 185.6 °C and with 20% mass loss in the TG curve. We suspected that the escape of the internal solvent of the compound was not a simple solvent volatilization, causing higher temperature than the boiling point of DMAC solvent. The product 2DEA-BTO had only one clear exothermic peak at 261.6 °C, which was about 20 °C higher than the decomposition temperature of the raw materials, indicating that the new compound showed excellent thermal stability. Meanwhile, the TG curve was decreased without obvious endothermic peaks, which indicated that the substance had sublimation properties. For the product NHA-BTO, the appearance of two endothermic peaks was mainly due to the evaporation of solvent methanol at 77.8 °C and the loss of crystalline water at 135.2 °C. More, the first decomposition peak of NHA-BTO was at 174.6 °C, also a clear exothermic peak appeared at 211.7 °C. At the same time, the thermal stabilities of the other five powder samples obtained during the screening were tested, and the results showed that their thermal behaviors were almost consistent with the raw material TKX-50.

### Crystal structure

**Fig. 4:**
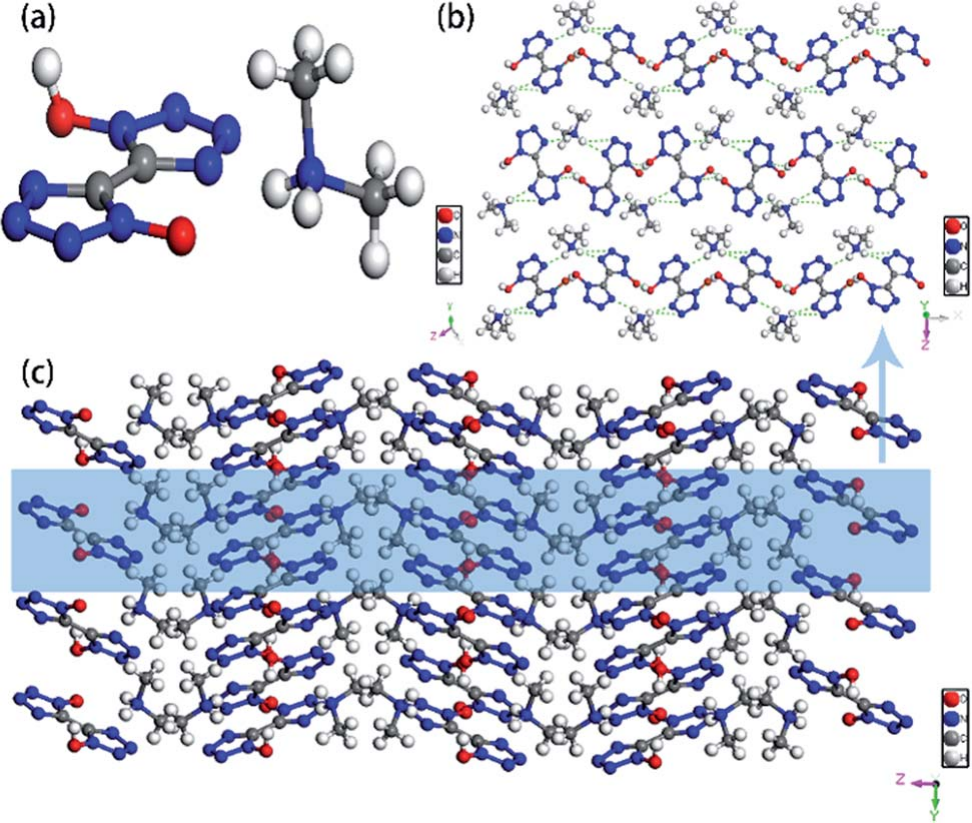
(a) The asymmetric unit of DMA-BTO; (b) the layer structure contained in DMA-BTO; (c) the 3D extended structure of DMA-BTO.

Colorless crystal of DMA-BTO ([NH_2_(CH_3_)_2_^+^][BTO^−^]) was a product obtained by recrystallization from DMAC solution, which was crystallized in orthorhombic *Pbca* space group and with the density of 1.529 g cm^−3^. This structure had been reported by Li *et al.*^35^ but we crystallized the same crystal structure in different solvents DMAC, and analyzed the crystal structure here briefly. There was a BTO^−^ anion and a DMA^+^ cation in the asymmetric unit of DMA-BTO (Fig. 4a). The anions and cations were connected by hydrogen bonds to form the one-dimensional chains like structures (Fig. 4b), these hydrogen bonds are as follow: N–H⋯N, N–H⋯O, O–H⋯O and O–H⋯N. The adjacent chains were arranged together to form a 2D layer, and then the layer structures were packed to form the 3D extended structure of DMA-BTO (Fig. 4c).

**Fig. 5:**
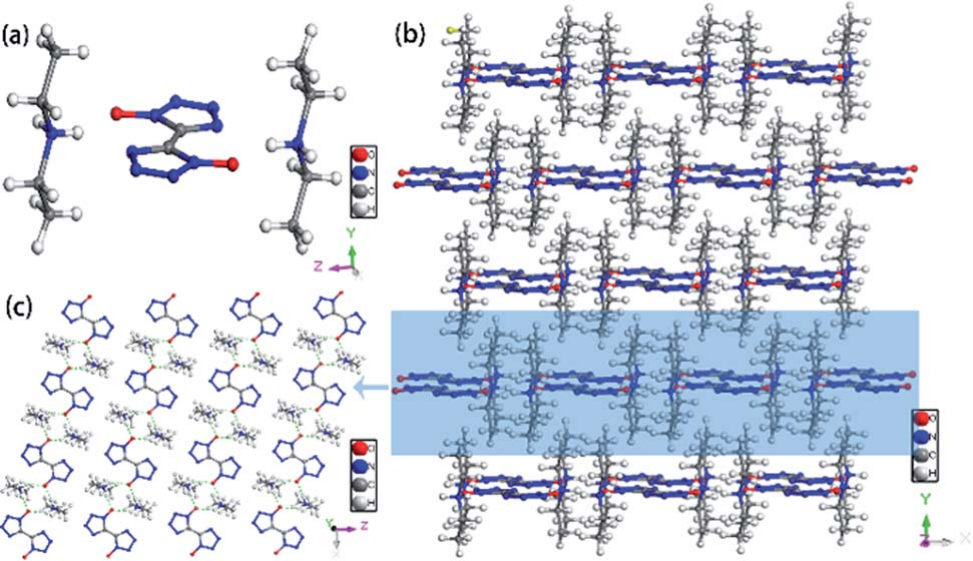
(a) The crystal structure of 2DEA-BTO; (b) the layer structure contained in 2DEA-BTO; (c) the 3D extended structure of 2DEA-BTO.

**Fig. 6:**
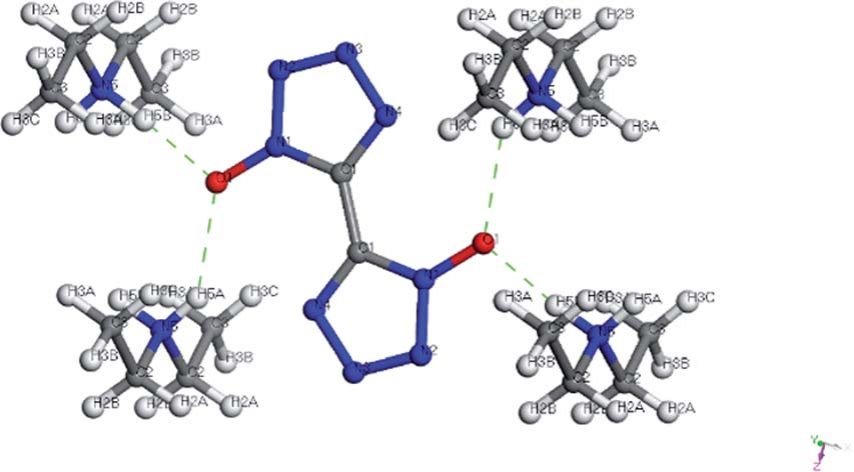
The hydrogen bonds of the product 2DEA-BTO.

Colorless crystal of 2DEA-BTO ([NH_2_(CH_3_CH_2_)_2_^+^]_2_[BTO^2−^]) was a product obtained by recrystallization from DEF solution, which was crystallized in monoclinic *C*2/*m* and with the density of 1.172 g cm^−3^. There was one BTO^2−^anion and two DEA^+^ cations in the asymmetric unit of 2DEA-BTO (Fig. 5a). Each BTO^2−^ anion was interacted with two adjacent DEA^+^ anions to form a linear structure running along *X*-axis direction *via* hydrogen bonds of NH⋯O, the adjacent linear structures were arranged with each other to form layer structures (Fig. 5b). In order to better understand the substance, we performed a detailed analysis of hydrogen bonding shown in Fig. 6. The type of hydrogen bonding was N–H⋯O and the bond length was in the range of 2.745–2.838 Å, which were listed in Table 3. Finally, the layer structures were packed into a 3D extended structure of [NH_2_(CH_3_CH_2_)_2_^+^]_2_[BTO^2−^] (Fig. 5c).

**Table 3:** Hydrogen bonds for 2DEA-BTO

D–H⋯A	*d*(D–H)	*d*(H⋯A)	*d*(D⋯A)	∠(DHA)
N(5)–H(5B)⋯O(1)	0.974	1.773	2.745	175.144
N(5)–H(5A)⋯O(1)	0.893	2.092	2.838	140.438

**Fig. 7:**
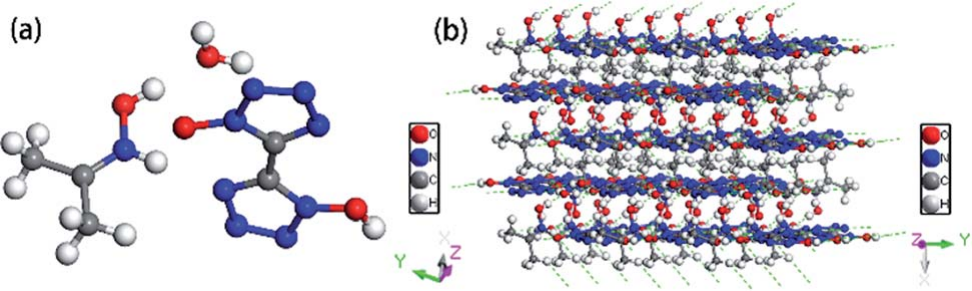
(a) The asymmetric unit of NHA-BTO; (b) the layer structure contained in NHA-BTO.

**Fig. 8:**
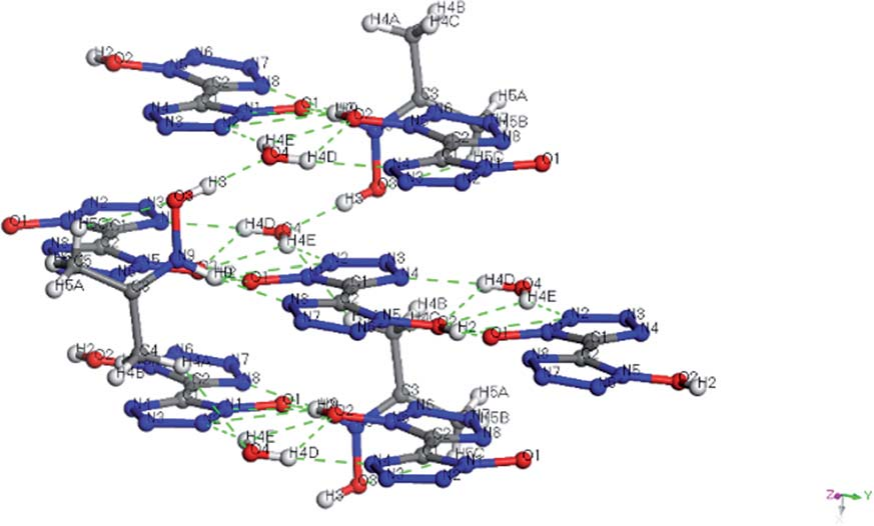
The hydrogen bonds of the product NHA-BTO.

Colorless crystal of NHA-BTO ([NHOH(CH_3_CCH_3_)^+^][BTO^−^]·H_2_O) was the product obtained by recrystallization from AC/MT mixed solution, which belonged to triclinic *P*1̄ space group and with the density of 1.560 g cm^−3^. The asymmetric unit of NHA-BTO contained a BTO^−^ anion, a NHOH(CH_3_CCH_3_)^+^ cation, and a water molecule shown in Fig. 7a. The 3D structure of NHA-BTO (Fig. 7b) was within five types of hydrogen bonds. To be specific, each BTO^−^ anion was interacted with adjacent BTO^−^ anions *via* hydrogen bonds of O–H⋯N, in which the bond length was in the range of 3.112–3.137 Å. Each BTO^−^ anion was interacted with adjacent NHOH(CH_3_CCH_3_)^+^ cation *via* hydrogen bonds of N–H⋯O and N–H⋯N, the bond lengths of N–H⋯O, N–H⋯N were 2.989 Å and 2.795 Å, respectively. Each BTO^−^ anion was interacted with two water molecule *via* the hydrogen bonds of O–H⋯N and O–H⋯O, the hydrogen bond lengths of O–H⋯N, O–H⋯O were in the range of 2.881–2.994 Å and 2.910 Å, respectively. Moreover, each NHOH(CH_3_CCH_3_)^+^ anion was interacted with a water molecule *via* hydrogen bonds of O–H⋯O, the bond length was 2.523 Å. Similarly, the hydrogen bonds contained in the structure were summarized in Fig. 8 and Table 4.

**Table 4:** Hydrogen bonds for 2DEA-BTO

D–H⋯A	*d*(D–H)	*d*(H⋯A)	*d*(D⋯A)	∠(DHA)
O(2)–H(2)⋯O(1)#1	0.946(18)	1.481(19)	2.4248(18)	175(3)
O(2)–H(2)⋯N(1)#1	0.946(18)	2.33(2)	3.1371(18)	143(3)
O(2)–H(2)⋯N(2)#1	0.946(18)	2.62(3)	3.112(2)	113(2)
C(4)–H(4A)⋯O(4)#2	0.99(3)	2.63(3)	3.448(3)	140(2)
N(9)–H(9)⋯O(1)	0.93(3)	2.47(3)	2.989(2)	115.6(19)
N(9)–H(9)⋯N(8)	0.93(3)	1.90(3)	2.795(2)	162(2)
O(3)–H(3)⋯O(4)	0.870(17)	1.653(18)	2.523(2)	178(3)
O(4)–H(4D)⋯N(4)#3	0.88(3)	2.00(3)	2.881(2)	175(3)
O(4)–H(4E)⋯O(2)#3	0.88(4)	2.42(3)	2.910(2)	116(3)
O(4)–H(4E)⋯N(2)#4	0.88(4)	2.16(4)	2.994(2)	158(3)

### Energetic properties

Detonation velocity (*D*) and detonation pressure (*P*) are important parameters for evaluating the explosion characteristics of high-energy materials. In order to verify the energy characteristics of the obtained crystals, the standard molar enthalpy of formation (Δ_f_*H*^θ^_m_) of compounds was calculated *via* Energetic Materials Studio 1.0 (ESI†), and the detonation velocity (*D*) and detonation pressure (*P*) of the new compounds were further calculated by EXPLO5 version 6.02 (ref. 37) and the results were shown in Table 5. Compared with the detonation velocity (9698 m s^−1^) and detonation pressure (42.4 GPa) of the raw material TKX-50, the detonation properties of three obtained compounds in this work were significantly reduced. Among these three compounds, compound DMA-BTO showed the highest detonation performance with the detonation velocity and detonation pressure values of 8358.1 m s^−1^ and 23.7 GPa, respectively. The 2DEA-BTO with the detonation velocity and detonation pressure values of 7135.6 m s^−1^ and 13.6 GPa, also the NHA-BTO with the detonation velocity and detonation pressure values of 7863.5 m s^−1^ and 21.4 GPa, respectively. In short, these new compounds have significantly destroyed the excellent performance of TKX-50. Although energetic ionic salts have been widely studied in the field of energetic materials and have many advantages, we also need to consider their solid form stability in the application of weapons.

**Table 5:** Energetic properties of TKX-50 and compounds 1 to 3

Compound	*ρ* a (g cm^−3^)	*T*_d_ b (K) °C	Δ_f_*H*^θ^_m_^c^ (kJ mol^−1^)	*P* d (GPa)	*D* e (m s^−1^)
TKX-50	1.918	293	446.6	42.4	9698
DMA-BTO	1.529	293	711.8	23.7	8358.1
2DEA-BTO	1.172	293	548.4	13.6	7135.6
NHA-BTO	1.560	293	690.2	21.4	7863.5

a Density measured by gas pycnometer for TKX-50 (25 °C) and SXRD for three new compounds.

b Decomposition temperature.

c Molar enthalpy of formation obtained by calculation.

d Detonation pressure (calculated with EXPLO5 v6.02).

e Detonation velocity (calculated with EXPLO 5 v6.02).

## Conclusions

In summary, we applied four crystallization methods to study the effect of different solvents on the structural evolution of energetic compound TKX-50. Several powder samples and three single crystal structures have been successfully screened. Among them, the compound [NH_2_(CH_3_)_2_^+^][BTO^−^] was obtained by crystallization in DMAC, the compound [NH_2_(CH_3_CH_2_)_2_^+^]_2_[BTO^2−^] was obtained in DEF, and the compound [NHOH(CH_3_CCH_3_)^+^][BTO^−^]·H_2_O was obtained in acetone/methyl alcohol mixed solvent. This work further demonstrates the possible evolution of TKX-50 during the induction of different solvent systems. Results showed that the use of three solvents (acetone/methyl alcohol, DMAC and DEF) would change the original structure of TKX-50. More important, the results obtained here could increase our knowledge of TKX-50 and reduce the security risks during future synthesis and storage.

## Conflicts of interest

There are no conflicts to declare.

## Supplementary Material

RA-010-D0RA01182G-s001

RA-010-D0RA01182G-s002
