# Supplementary material for: Crystal structure evolution of an energetic compound dihydroxylammonium 5,5′-bistetrazole-1,1′-diolate induced by solvents
Source: RSC Adv. 2020 Mar 26;10(20):11939–44. doi: 10.1039/d0ra01182g (PMC9050641; doi:10.1039/d0ra01182g)
Supplement: RA-010-D0RA01182G-s001 [file RA-010-D0RA01182G-s001.pdf]

Electronic Supplementary Material (ESI) for RSC Advances.
This journal is © The Royal Society of Chemistry 2020

Electronic Supplementary Information

Crystal structure evolution of an energetic compound dihydroxylammonium 5,5′-bistetrazole-1,1′-diolate induced by solvents

Xin Xu,[a,b] Dong Chen,[a] Hongzhen Li,[a] Mi Yan,[a] Ying Xiong,[a] Haixia Zhao*[b] and Rong Xu,*[a]

[a] Institute of Chemical Materials, China Academy of Engineering Physics, Mianyang, 621900, China

[b] College of Environment and Safety Engineering, North University of China, Taiyuan, 030051, China

Corresponding author: E-mail: xurwjy@caep.cn (Rong Xu)
zhhx@nuc.edu.cn (Haixia Zhao)

Table of Contents:

1.1 Materials.
1.2 Single Crystal X-ray Diffraction (SC-XRD).
1.3 DSC analysis.
1.4 Structural data and dehydration of 2DEA-BTO.
1.5 Structural data and dehydration of DMA-BTO (recrystallization from DMF solution)
1.6 Structural data and dehydration of DMA-BTO (recrystallization from DMAC solution)
1.7 Structural data and dehydration of NHA-BTO
1.8 Hydrogen Bonding.
1.9 Standard molar enthalpy of formation ($\Delta_c H^0_m$).
2.0 PXRD
2.1 Elemental analysis
References

1.1 Materials.

TKX-50 was synthesized according to the reported method1. All solvents used for crystallization were of analytical grade and purchased from commercial suppliers and were used as received.

1.2 Single Crystal X-ray Diffraction (SC-XRD).

Suitable crystals were chosen and placed in a Rigaku supernova Single X-ray Diffractometer area detector using graphite monochromated Mo Kα radiation (λ= 0.71073 Å) at 293(2) K. Its structures were solved by direct methods using OLEX2 and refined by fullmatrix least-squares on F2 (SHELXL-2013). All non-hydrogen atoms were anisotropically refined. Hydrogen atoms attached to oxygen were placed from difference Fourier maps and were refined using riding model. Data collection parameters and refinement statistics were given in Table 1.

Table 1Basic crystallographic Data of DEF$_C$ DMF$_C$ and DMAC$_C$

	2DEA-BTO	DMA-BTO	DMA-BTO	NHA-BTO
chemical formula	$C_{10}H_{24}N_{10}O_2$	$C_4H_9N_9O_2$	$C_4H_9N_9O_2$	$C_5H_{11}N_9O_4$
molecular weight	316.39	215.20	215.20	261.23
Crystal system	Monoclinic	Orthorhombic	Orthorhombic	Triclinic
Space group	C 2/m	P b c a	P b c a	P -1
T(K)	293(2) K	293(2) K	296(2) K	293(2) K
a(Å)	8.3308(10)	11.6746(5)	11.6722(4)	6.8110(5)

b(Å)	12.0024(16)	8.8149(3)	8.8122(4)	7.3961(5)
c(Å)	9.2986(12)	18.1710(7)	18.1686(6)	11.6431(9)
α(deg)	90	90	90	90
β(deg)	105.393(3)	90	90	95.114(2)
γ(deg)	90	90	90	90
V(Å^3)	896.4(2)	1869.99(13)	1868.78(12)	556.19(7)
Z	2	8	8	2
D_{calcd}(g cm^{-3})	1.172	1.529	1.530	1.560
F (000)	340	896	896	272
μ(mm^{-1})	0.087	0.125	0.125	0.133
θ range (deg)	3.052-25.996	2.841-25.999	2.841-25.999	2.881-25.999
Reflections collected	7344	8575	8539	7339
Index ranges	-10≦h≦10<br>-14≦k≦14<br>-11≦l≦11	-14≦h≦14,<br>-9≦k≦10,<br>-22≦l≦18	-13≦h≦14,<br>-6≦k≦10,<br>-22≦l≦21	-8≦h≦8,<br>-9≦k≦8,<br>-13≦l≦14
Data/restraints	924 / 0	1825 / 1	1820 / 1	2180 / 2
parameters	88	151	151	208
Final R index [I >2σ(I)]	R1=0.0402,W R2=0.1111	R1 = 0.0385, WR2 = 0.0952	R1 = 0.0361, WR2 = 0.0913	R1 = 0.0422, WR2 = 0.1001
Final R index [all data]	R1=0.0492,W R2=0.1183	R1 = 0.0484, WR2 = 0.1032	R1 = 0.0452, WR2 = 0.0986	R1 = 0.0551, WR2 = 0.1103
GooF	1.083	1.057	1.069	1.065

1.3 DSC analysis.

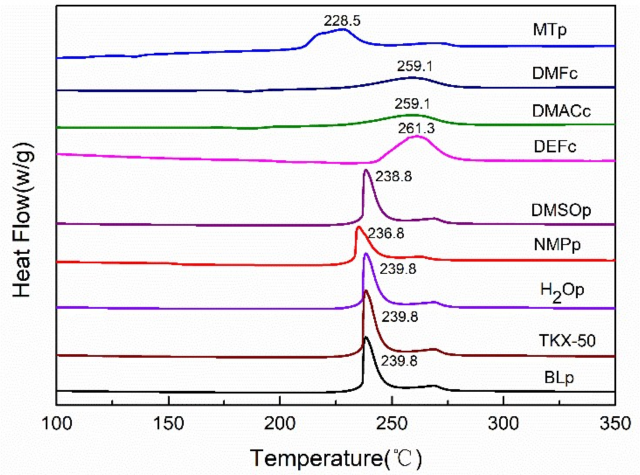

Figure 1. DSC curves of TKX-50 and the results by experiment.

The differential scanning calorimetry (DSC, Mettler Toledo) was performed to record the thermal behaviors of the raw materials and different morphologies crystals from 50 to 350°C. The sample (0.2 mg) was heated at a heating rate of 10°C/min with a stream of flowing nitrogen at 20mL/min. Three replicates of each sample.

The DSC curves of BLp, H2Op, NMPp and DMSOp are basically consistent with the raw material TKX-50, all of which have only one decomposition peak, and the main decomposition temperatures are not much different. However, the main decomposition peak of MTp is different from the raw material and decomposes earlier than the raw material.

Combining the DSC curves of DMFc, DMACc, DEFc, H2Op, NMPp, DMSOp, MTp, and BLp with TKX-50, it can be found that the decomposition trends of four compounds and TKX-50 and the decomposition temperature of the main decomposition peak are different, indicating that TKX

The -50 changed and new solid matter was created in these solvents.

1.4 Structural data and dehydration of 2DEA-BTO.

Table 2. Bond lengths [Å] and angles [°] for DEF

Bond lengths	Bond angles	Bond lengths	Bond angles
O(1)-N(1)	1.307(2)	O(1)-N(1)-C(1)	129.13(16)
N(1)-C(1)	1.338(2)	O(1)-N(1)-N(2)	122.09(14)
N(1)-N(2)	1.339(2)	C(1)-N(1)-N(2)	108.78(15)
N(2)-N(3)	1.309(2)	N(3)-N(2)-N(1)	106.18(15)
N(3)-N(4)	1.344(2)	N(2)-N(3)-N(4)	110.66(16)
N(4)-C(1)	1.325(2)	C(1)-N(4)-N(3)	106.25(15)
C(1)-C(1)#1	1.443(4)	N(4)-C(1)-N(1)	108.13(16)
N(5)-C(2)#2	1.477(2)	N(4)-C(1)-C(1)#1	127.67(19)
N(5)-C(2)	1.477(2)	N(1)-C(1)-C(1)#1	124.2(2)
N(5)-H(5A)	0.89(2)	C(2)#2-N(5)-C(2)	113.1(2)
N(5)-H(5B)	0.97(2)	C(2)#2-N(5)-H(5A)	110.3(7)
C(2)-C(3)	1.487(4)	C(2)-N(5)-H(5A)	110.3(7)
C(2)-H(2A)	0.98(2)	C(2)#2-N(5)-H(5B)	108.5(6)
C(2)-H(2B)	0.99(2)	C(2)-N(5)-H(5B)	108.5(6)
C(3)-H(3A)	1.00(4)	H(5A)-N(5)-H(5B)	106(2)
C(3)-H(3B)	1.02(4)	N(5)-C(2)-C(3)	110.7(2)
C(3)-H(3C)	0.93(4)	N(5)-C(2)-H(2A)	108.0(13)
C(3)-C(2)-H(2A)	108.3(13)	C(2)-C(3)-H(3B)	108.2(19)
N(5)-C(2)-H(2B)	109.8(14)	H(3A)-C(3)-H(3B)	106(2)
C(3)-C(2)-H(2B)	106.9(14)	C(2)-C(3)-H(3C)	110(3)
H(2A)-C(2)-H(2B)	113(2)	H(3A)-C(3)-H(3C)	109(3)
C(2)-C(3)-H(3A)	108.6(18)	H(3B)-C(3)-H(3C)	114(3)

Table 3. Torsion angles/° for 2DEA-BTO

O(1)-N(1)-N(2)-N(3)	180.000(1)
C(1)-N(1)-N(2)-N(3)	0.000(1)
N(1)-N(2)-N(3)-N(4)	0.000(1)
N(2)-N(3)-N(4)-C(1)	0.000(1)
N(3)-N(4)-C(1)-N(1)	0.000(1)
N(3)-N(4)-C(1)-C(1)#1	180.000(1)
O(1)-N(1)-C(1)-N(4)	180.000(1)
N(2)-N(1)-C(1)-N(4)	0.000(1)
O(1)-N(1)-C(1)-C(1)#1	0.000(1)
N(2)-N(1)-C(1)-C(1)#1	180.000(1)
C(2)#2-N(5)-C(2)-C(3)	-179.6(2)

1.5 Structural data and dehydration of DMA-BTO

Table 4. Bond lengths [Å] and angles [°] for DMA-BTO

Bond lengths	Bond angles	Bond lengths	Bond angles
N(1)-O(1)	1.3225(14)	O(1)-N(1)-N(2)	121.82(11)
N(1)-N(2)	1.3301(16)	O(1)-N(1)-C(1)	128.95(11)
N(1)-C(1)	1.3328(17)	N(2)-N(1)-C(1)	109.23(11)
N(2)-N(3)	1.3038(18)	N(3)-N(2)-N(1)	106.13(11)
N(3)-N(4)	1.3452(18)	N(2)-N(3)-N(4)	110.79(11)
N(4)-C(1)	1.3259(17)	C(1)-N(4)-N(3)	105.85(12)
N(5)-C(2)	1.3218(16)	C(2)-N(5)-N(6)	105.98(11)
N(5)-N(6)	1.3542(16)	N(7)-N(6)-N(5)	111.07(11)
N(6)-N(7)	1.2957(18)	N(6)-N(7)-N(8)	105.66(11)
N(7)-N(8)	1.3346(18)	C(2)-N(8)-N(7)	109.81(11)
N(8)-C(2)	1.3355(16)	C(2)-N(8)-O(2)	129.19(11)
N(8)-O(2)	1.3476(14)	N(7)-N(8)-O(2)	120.96(11)
N(9)-C(4)	1.467(2)	C(4)-N(9)-C(3)	113.00(13)
N(9)-C(3)	1.473(2)	C(4)-N(9)-H(9A)	108.0(12)
N(9)-H(9A)	0.875(19)	C(3)-N(9)-H(9A)	109.3(12)
N(9)-H(9B)	0.90(2)	C(4)-N(9)-H(9B)	108.3(12)
O(2)-H(2)	0.922(16)	C(3)-N(9)-H(9B)	110.4(12)
C(1)-C(2)	1.4481(18)	H(9A)-N(9)-H(9B)	107.7(16)
C(3)-H(3A)	0.9600	N(8)-O(2)-H(2)	103.9(13)
C(3)-H(3B)	0.9600	N(4)-C(1)-N(1)	108.00(11)
C(3)-H(3C)	0.9600	N(4)-C(1)-C(2)	127.68(12)
C(4)-H(4A)	0.9600	N(1)-C(1)-C(2)	124.32(11)
C(4)-H(4B)	0.9600	N(5)-C(2)-N(8)	107.48(11)
C(4)-H(4C)	0.9600	N(5)-C(2)-C(1)	127.52(12)
N(8)-C(2)-C(1)	125.00(12)	H(3A)-C(3)-H(3C)	109.5
N(9)-C(3)-H(3A)	109.5	H(3B)-C(3)-H(3C)	109.5
N(9)-C(3)-H(3B)	109.5	N(9)-C(4)-H(4A)	109.5
H(3A)-C(3)-H(3B)	109.5	N(9)-C(4)-H(4B)	109.5
N(9)-C(3)-H(3C)	109.5	H(4A)-C(4)-H(4B)	109.5
N(9)-C(4)-H(4C)	109.5	H(4A)-C(4)-H(4C)	109.5
H(4B)-C(4)-H(4C)	109.5		

Table 5. Torsion angles/° for DMA-BTO

O(1)-N(1)-N(2)-N(3)	-179.65(12)
C(1)-N(1)-N(2)-N(3)	-0.29(16)
N(1)-N(2)-N(3)-N(4)	0.46(18)
N(2)-N(3)-N(4)-C(1)	-0.45(18)

C(2)-N(5)-N(6)-N(7)	0.41(17)
N(5)-N(6)-N(7)-N(8)	-0.13(17)
N(6)-N(7)-N(8)-C(2)	-0.20(16)
N(6)-N(7)-N(8)-O(2)	-178.19(12)
N(3)-N(4)-C(1)-N(1)	0.26(16)
N(3)-N(4)-C(1)-C(2)	179.65(14)
O(1)-N(1)-C(1)-N(4)	179.32(13)
N(2)-N(1)-C(1)-N(4)	0.02(16)
O(1)-N(1)-C(1)-C(2)	-0.1(2)
N(2)-N(1)-C(1)-C(2)	-179.40(13)
N(6)-N(5)-C(2)-N(8)	-0.51(15)
N(6)-N(5)-C(2)-C(1)	179.34(13)
N(7)-N(8)-C(2)-N(5)	0.45(16)
O(2)-N(8)-C(2)-N(5)	178.23(13)
N(7)-N(8)-C(2)-C(1)	-179.41(13)
O(2)-N(8)-C(2)-C(1)	-1.6(2)
N(4)-C(1)-C(2)-N(5)	-178.82(14)
N(1)-C(1)-C(2)-N(5)	0.5(2)
N(4)-C(1)-C(2)-N(8)	1.0(2)
N(1)-C(1)-C(2)-N(8)	-179.69(12)

1.6 Structural data and dehydration of DMA-BTO

Table 6. Bond lengths [Å] and angles [°] for DMA-BTO

Bond lengths	Bond angles	Bond lengths	Bond angles
O(1)-N(1)	1.3471(16)	N(1)-O(1)-H(1)	105.1(15)
O(1)-H(1)	0.906(16)	N(2)-N(1)-C(1)	109.74(12)
O(2)-N(5)	1.3224(15)	N(2)-N(1)-O(1)	121.05(12)
N(1)-N(2)	1.337(2)	C(1)-N(1)-O(1)	129.18(13)
N(1)-C(1)	1.3371(18)	N(3)-N(2)-N(1)	105.69(12)
N(2)-N(3)	1.291(2)	N(2)-N(3)-N(4)	111.28(13)
N(3)-N(4)	1.3562(18)	C(1)-N(4)-N(3)	105.86(12)
N(4)-C(1)	1.3233(18)	O(2)-N(5)-C(2)	129.20(12)
N(5)-C(2)	1.3325(18)	O(2)-N(5)-N(6)	121.64(12)
N(5)-N(6)	1.3351(18)	C(2)-N(5)-N(6)	109.15(12)
N(6)-N(7)	1.2997(19)	N(7)-N(6)-N(5)	105.99(13)
N(7)-N(8)	1.346(2)	N(6)-N(7)-N(8)	111.07(13)
N(8)-C(2)	1.3260(18)	C(2)-N(8)-N(7)	105.74(13)
N(9)-C(4)	1.469(2)	C(4)-N(9)-C(3)	112.93(14)
N(9)-C(3)	1.474(2)	C(4)-N(9)-H(9A)	110.3(13)

N(9)-H(9A)	0.89(2)	C(3)-N(9)-H(9A)	107.2(13)
N(9)-H(9B)	0.90(2)	C(4)-N(9)-H(9B)	106.8(13)
C(1)-C(2)	1.445(2)	C(3)-N(9)-H(9B)	111.1(13)
C(3)-H(3A)	0.9600	H(9A)-N(9)-H(9B)	108.4(19)
C(3)-H(3B)	0.9600	N(4)-C(1)-N(1)	107.43(13)
C(3)-H(3C)	0.9600	N(4)-C(1)-C(2)	127.54(13)
C(4)-H(4A)	0.9600	N(1)-C(1)-C(2)	125.03(13)
C(4)-H(4B)	0.9600	N(8)-C(2)-N(5)	108.05(13)
C(4)-H(4C)	0.9600	N(8)-C(2)-C(1)	127.77(13)
N(5)-C(2)-C(1)	124.18(13)	H(3A)-C(3)-H(3B)	109.5
N(9)-C(3)-H(3A)	109.5	N(9)-C(3)-H(3C)	109.5
N(9)-C(3)-H(3B)	109.5	H(3A)-C(3)-H(3C)	109.5
H(3B)-C(3)-H(3C)	109.5	N(9)-C(4)-H(4C)	109.5
N(9)-C(4)-H(4A)	109.5	H(4A)-C(4)-H(4C)	109.5
N(9)-C(4)-H(4B)	109.5	H(4B)-C(4)-H(4C)	109.5
H(4A)-C(4)-H(4B)	109.5		

Table 7. Torsion angles/° for DMA-BTO

C(1)-N(1)-N(2)-N(3)	-0.05(17)
O(1)-N(1)-N(2)-N(3)	-178.25(13)
N(1)-N(2)-N(3)-N(4)	-0.13(18)
N(2)-N(3)-N(4)-C(1)	0.26(17)
O(2)-N(5)-N(6)-N(7)	-179.69(13)
C(2)-N(5)-N(6)-N(7)	-0.46(17)
N(5)-N(6)-N(7)-N(8)	0.54(19)
N(6)-N(7)-N(8)-C(2)	-0.41(19)
N(3)-N(4)-C(1)-N(1)	-0.28(16)
N(3)-N(4)-C(1)-C(2)	179.25(14)
N(2)-N(1)-C(1)-N(4)	0.22(16)
O(1)-N(1)-C(1)-N(4)	178.22(13)
N(2)-N(1)-C(1)-C(2)	-179.33(13)
O(1)-N(1)-C(1)-C(2)	-1.3(2)
N(7)-N(8)-C(2)-N(5)	0.11(17)
N(7)-N(8)-C(2)-C(1)	179.80(15)
O(2)-N(5)-C(2)-N(8)	179.37(13)
N(6)-N(5)-C(2)-N(8)	0.22(16)
O(2)-N(5)-C(2)-C(1)	-0.3(2)
N(6)-N(5)-C(2)-C(1)	-179.49(13)
N(4)-C(1)-C(2)-N(8)	-178.84(14)

N(1)-C(1)-C(2)-N(8)	0.6(2)
N(4)-C(1)-C(2)-N(5)	0.8(2)
N(1)-C(1)-C(2)-N(5)	-179.74(13)

1.6 Structural data and dehydration of NHA-BTO

Table 8. Bond lengths [Å] and angles [°] for NHA-BTO

O(1)-N(1)	1.3330(18)	O(3)-N(9)	1.362(2)
O(2)-N(5)	1.3247(18)	O(3)-H(3)	0.870(17)
O(2)-H(2)	0.946(18)	N(9)-C(3)	1.268(2)
N(1)-N(2)	1.332(2)	N(9)-H(9)	0.93(3)
N(1)-C(1)	1.332(2)	C(3)-C(5)	1.475(3)
N(2)-N(3)	1.303(2)	C(3)-C(4)	1.476(3)
N(3)-N(4)	1.347(2)	C(4)-H(4A)	0.99(3)
N(4)-C(1)	1.328(2)	C(4)-H(4B)	1.01(3)
N(5)-C(2)	1.331(2)	C(4)-H(4C)	0.91(3)
N(5)-N(6)	1.334(2)	C(5)-H(5A)	0.92(3)
N(6)-N(7)	1.301(2)	C(5)-H(5B)	0.94(4)
N(7)-N(8)	1.344(2)	C(5)-H(5C)	0.93(3)
N(8)-C(2)	1.324(2)	O(4)-H(4D)	0.88(3)
C(1)-C(2)	1.444(2)	O(4)-H(4E)	0.88(4)
N(5)-O(2)-H(2)	108(2)	N(2)-N(3)-N(4)	110.50(14)
N(2)-N(1)-O(1)	123.11(14)	C(1)-N(4)-N(3)	106.31(14)
N(2)-N(1)-C(1)	109.57(14)	O(2)-N(5)-C(2)	128.10(14)
O(1)-N(1)-C(1)	127.32(15)	O(2)-N(5)-N(6)	122.49(14)
N(3)-N(2)-N(1)	106.16(14)	C(2)-N(5)-N(6)	109.39(14)
N(7)-N(6)-N(5)	106.10(14)	N(8)-C(2)-C(1)	126.59(14)
N(6)-N(7)-N(8)	110.51(14)	N(5)-C(2)-C(1)	125.83(15)
C(2)-N(8)-N(7)	106.42(14)	N(9)-O(3)-H(3)	107.3(18)
N(4)-C(1)-N(1)	107.47(15)	C(3)-N(9)-O(3)	121.40(16)
N(4)-C(1)-C(2)	128.63(15)	C(3)-N(9)-H(9)	120.3(16)
N(1)-C(1)-C(2)	123.91(15)	O(3)-N(9)-H(9)	118.2(16)
N(8)-C(2)-N(5)	107.58(15)	N(9)-C(3)-C(5)	121.4(2)
N(9)-C(3)-C(4)	117.07(18)	H(4B)-C(4)-H(4C)	103(2)
C(5)-C(3)-C(4)	121.6(2)	C(3)-C(5)-H(5A)	107.7(19)
C(3)-C(4)-H(4A)	111.3(15)	C(3)-C(5)-H(5B)	109.8(19)
C(3)-C(4)-H(4B)	110.8(15)	H(5A)-C(5)-H(5B)	109(3)
H(4A)-C(4)-H(4B)	110(2)	C(3)-C(5)-H(5C)	109.4(17)
C(3)-C(4)-H(4C)	107.9(19)	H(5A)-C(5)-H(5C)	111(3)
H(4A)-C(4)-H(4C)	114(2)	H(5B)-C(5)-H(5C)	110(3)
H(4D)-O(4)-H(4E)	108(3)		

Table 7. Torsion angles/° for NHA-BTO

O(1)-N(1)-N(2)-N(3)	-179.25(17)
C(1)-N(1)-N(2)-N(3)	-0.2(2)
N(1)-N(2)-N(3)-N(4)	0.2(2)
N(2)-N(3)-N(4)-C(1)	-0.1(2)
O(2)-N(5)-N(6)-N(7)	-178.92(16)
C(2)-N(5)-N(6)-N(7)	-0.4(2)
N(5)-N(6)-N(7)-N(8)	0.3(2)
N(6)-N(7)-N(8)-C(2)	-0.1(2)
N(3)-N(4)-C(1)-N(1)	-0.1(2)
N(3)-N(4)-C(1)-C(2)	179.80(18)
N(2)-N(1)-C(1)-N(4)	0.2(2)
O(1)-N(1)-C(1)-N(4)	179.16(17)
N(2)-N(1)-C(1)-C(2)	-179.69(16)
O(1)-N(1)-C(1)-C(2)	-0.7(3)
N(7)-N(8)-C(2)-N(5)	-0.2(2)
N(7)-N(8)-C(2)-C(1)	179.99(17)
O(2)-N(5)-C(2)-N(8)	178.78(17)
N(6)-N(5)-C(2)-N(8)	0.4(2)
O(2)-N(5)-C(2)-C(1)	-1.4(3)
N(6)-N(5)-C(2)-C(1)	-179.81(16)
N(4)-C(1)-C(2)-N(8)	170.46(18)
N(1)-C(1)-C(2)-N(8)	-9.7(3)
N(4)-C(1)-C(2)-N(5)	-9.3(3)
N(1)-C(1)-C(2)-N(5)	170.52(17)
O(3)-N(9)-C(3)-C(5)	1.2(3)
O(3)-N(9)-C(3)-C(4)	179.87(18)

1.8 Hydrogen Bonding.

Table 10 Hydrogen bond lengths /Å and angles /° of DMA-BTO.

D-H...A	d(D-H)	d(H...A)	d(D...A)	<(DHA)
N(9)-H(9A)...N(7)#1	0.89(2)	2.65(2)	3.373(2)	139.9(17)
N(9)-H(9A)...N(8)#1	0.89(2)	2.08(2)	2.956(2)	168.8(19)
N(9)-H(9B)...O(2)	0.90(2)	2.37(2)	2.8456(18)	113.2(15)
N(9)-H(9B)...N(4)	0.90(2)	2.09(2)	2.933(2)	155.9(18)
O(1)-H(1)...O(2)#2	0.906(16)	1.554(17)	2.4578(16)	175(2)
O(1)-H(1)...N(5)#2	0.906(16)	2.346(18)	3.1770(16)	152(2)

Table 11 Hydrogen bond lengths /Å and angles /° of DMA-BTO. (DMF solution)

D-H...A	d(D-H)	d(H...A)	d(D...A)	<(DHA)
O(2)-H(2)...N(1)#1	0.922(16)	2.328(17)	3.1748(16)	152.5(18)
O(2)-H(2)...N(2)#1	0.922(16)	2.700(19)	3.3285(17)	126.2(16)
O(2)-H(2)...O(1)#1	0.922(16)	1.537(16)	2.4567(15)	175(2)
N(9)-H(9A)...N(3)#2	0.875(19)	2.673(19)	3.3710(18)	137.6(15)
N(9)-H(9A)...N(4)#2	0.875(19)	2.10(2)	2.9587(18)	166.9(17)
N(9)-H(9B)...N(5)	0.90(2)	2.09(2)	2.9338(18)	154.1(16)
N(9)-H(9B)...O(1)	0.90(2)	2.365(18)	2.8466(16)	113.2(14)

Table 12 Hydrogen bond lengths /Å and angles /° of NHA-BTO.

D-H...A	d(D-H)	d(H...A)	d(D...A)	<(DHA)
O(2)-H(2)...O(1)#1	0.946(18)	1.481(19)	2.4248(18)	175(3)
O(2)-H(2)...N(1)#1	0.946(18)	2.33(2)	3.1371(18)	143(3)
O(2)-H(2)...N(2)#1	0.946(18)	2.62(3)	3.112(2)	113(2)
C(4)-H(4A)...O(4)#2	0.99(3)	2.63(3)	3.448(3)	140(2)
N(9)-H(9)...O(1)	0.93(3)	2.47(3)	2.989(2)	115.6(19)
N(9)-H(9)...N(8)	0.93(3)	1.90(3)	2.795(2)	162(2)
O(3)-H(3)...O(4)	0.870(17)	1.653(18)	2.523(2)	178(3)
O(4)-H(4D)...N(4)#3	0.88(3)	2.00(3)	2.881(2)	175(3)
O(4)-H(4E)...O(2)#3	0.88(4)	2.42(3)	2.910(2)	116(3)

1.9 Standard molar enthalpy of formation ($\Delta_c H^0{}_m$).

The Energy Materials Studio 1.0, which is an integrated calculation system designed by ICM (CAEP), was applied to calculate the entalpy of formation of new compounds. The atomization method was used to calculate the gas phase enthalpies of formation for anion and cation with CBS-4M level, and the lattice energy was calculated through PBE functional and TS dispersion correction with Norm conserving pseudopotential (energy cutoff of 700eV) generalization and TS dispersion correction.[1-3]

2.0 PXRD

Powder X-ray diffraction patterns were recorded with the Cu- Kα radiation (λ= 1.54056 Å). The current and voltage were set at 30 mA and 40 KV, respectively. The data were collected over the range from 5◦ to 50◦ with a step size of 0.02°. Three new compounds and TKX-50 PXRD based on the Cambridge Crystal Structure Database are shown in Figure 2.

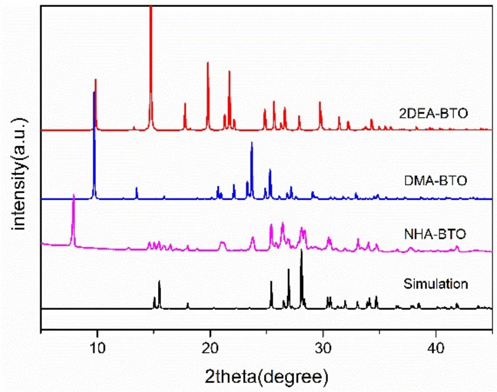

Figure 2 Three new compounds and TKX-50 simulated patterns

2.1 Elemental analysis

1) Elemental analysis calcd (%) for $C_4H_9N_9O_2$ (DMA-BTO) (215): C (22.3), H (4.18), N (58.6); found: C (22.34), H (4.08), N (59.2).

2) Elemental analysis calcd (%) for $C_{10}H_{24}N_{10}O_2$ (2DEA-BTO) (316): C (37.97), H (7.59), N (44.3); found: C (37.8), H (7.48), N (43.9).

3) Elemental analysis calcd (%) for $C_5H_{11}N_9O_4$ (NHA-BTO) (261): C (22.98), H (4.21), N (48.27); found: C (23.12), H (4.15), N (48.12).

References

1. N. Fischer, D. Fischer, T. M. Klapotke, D. J. Piercey, J. Stierstorfer, *J. Mater. Chem.,* 2012, **22,** 20418–20422.
2. M. J.Frisch, et al, Gaussian 09, Revision A.02, Gaussian, Inc., Wallingford CT, 2009
3. N.Fischer, T. M. Klapötke, J. Stierstorfer, Wiedemann, C. Polyhedron 2011, **30,** 2374–2386.
